# Liquid versus gel handrub formulation: a prospective intervention study

**DOI:** 10.1186/cc5906

**Published:** 2007-05-03

**Authors:** Ousmane Traore, Stéphane Hugonnet, Jann Lübbe, William Griffiths, Didier Pittet

**Affiliations:** 1 Infection Control Programme, University of Geneva Hospitals, 24 Rue Micheli-du-Crest, 1211 Geneva 14, Switzerland; 2 Service d'Hygiène Hospitalière, Hôpital Gabriel Montpied, CHU de Clermont-Ferrand, 56 Rue Montalembert, 63003 Clermont-Ferrand cedex 1, France; 3 Service of Dermatology, University of Geneva Hospitals, 24 Rue Micheli-du-Crest, 1211 Geneva 14, Switzerland; 4 Hospital Pharmacy, University of Geneva Hospitals, 24 Rue Micheli-du-Crest, 1211 Geneva 14, Switzerland

## Abstract

**Introduction:**

Hand hygiene is one of the cornerstones of the prevention of health care-associated infection, but health care worker (HCW) compliance with good practices remains low. Alcohol-based handrub is the new standard for hand hygiene action worldwide and usually requires a system change for its successful introduction in routine care. Product acceptability by HCWs is a crucial step in this process.

**Methods:**

We conducted a prospective intervention study to compare the impact on HCW compliance of a liquid (study phase I) versus a gel (phase II) handrub formulation of the same product during daily patient care. All staff (102 HCWs) of the medical intensive care unit participated. Compliance with hand hygiene was monitored by a single observer. Skin tolerance and product acceptability were assessed using subjective and objective scoring systems, self-report questionnaires, and biometric measurements. Logistic regression was used to estimate the association between predictors and compliance with the handrub formulation as the main explanatory variable and to adjust for potential risk factors.

**Results:**

Overall compliance (phases I and II) with hand hygiene practices among nurses, physicians, nursing assistants, and other HCWs was 39.1%, 27.1%, 31.1%, and 13.9%, respectively (*p *= 0.027). Easy access to handrub improved compliance (35.3% versus 50.6%, *p *= 0.035). Nurse status, working on morning shifts, use of the gel formulation, and availability of the alcohol-based handrub in the HCW's pocket were independently associated with higher compliance. Immediate accessibility was the strongest predictor. Based on self-assessment, observer assessment, and the measurement of epidermal water content, the gel performed significantly better than the liquid formulation.

**Conclusion:**

Facilitated access to an alcohol-based gel formulation leads to improved compliance with hand hygiene and better skin condition in HCWs.

## Introduction

Health care workers' (HCWs') hands play a key role in the patient-to-patient transmission of microbial pathogens, and hand hygiene is the primary measure to prevent cross-infection in hospitals [1]. Improvement in hand hygiene practices reduces health care-associated infection [2] and the burden of disease in the community [3,4]. However, the impact of hand hygiene in reducing infections relies on multiple factors, including the type of hand-cleansing agent used [1,2]. Hand antisepsis with alcohol-based handrubs has many advantages over handwashing with soap and water: it requires less time, acts faster, and is more efficacious, more convenient, and better tolerated by HCWs' skin [2].

Studies have shown that handrubbing contributes to enhanced compliance [5-7]. However, the use of a product also depends on dermal tolerance and user acceptability with consideration of parameters such as fragrance, drying speed, and skin feeling following application [8,9]. It has been suggested that among alcohol-based handrubs, gels could be associated with better skin care properties and dermal tolerance than liquid formulations, thus leading to more acceptable products and to potentially better compliance [10-13]. To our knowledge, there is no published study suggesting that adherence is higher when using gels rather than liquid formulations. We aimed to assess whether the introduction of a gel formulation would result in increased compliance with hand hygiene. A secondary objective was to compare the user acceptability and skin tolerance of the two formulations.

## Materials and methods

### Study design

The intervention study was conducted in the medical intensive care unit (ICU) of the University of Geneva Hospitals (Geneva, Switzerland), a 2,300-bed, tertiary care institution serving a population of approximately 800,000. The 18-bed unit includes coronary care beds and admits approximately 1,500 patients per year for a mean length of stay of four days. In 2004, the mean admission APACHE (Acute Physiology and Chronic Health Evaluation) score was 14 and approximately 40% of the critically ill patients (excluding the coronary care patients) received mechanical ventilation. The median 24-hour nurse-to-patient ratio was 2.2 in 2004 and did not differ between the study phases. The institutional review board approved the study.

**Figure 1:**
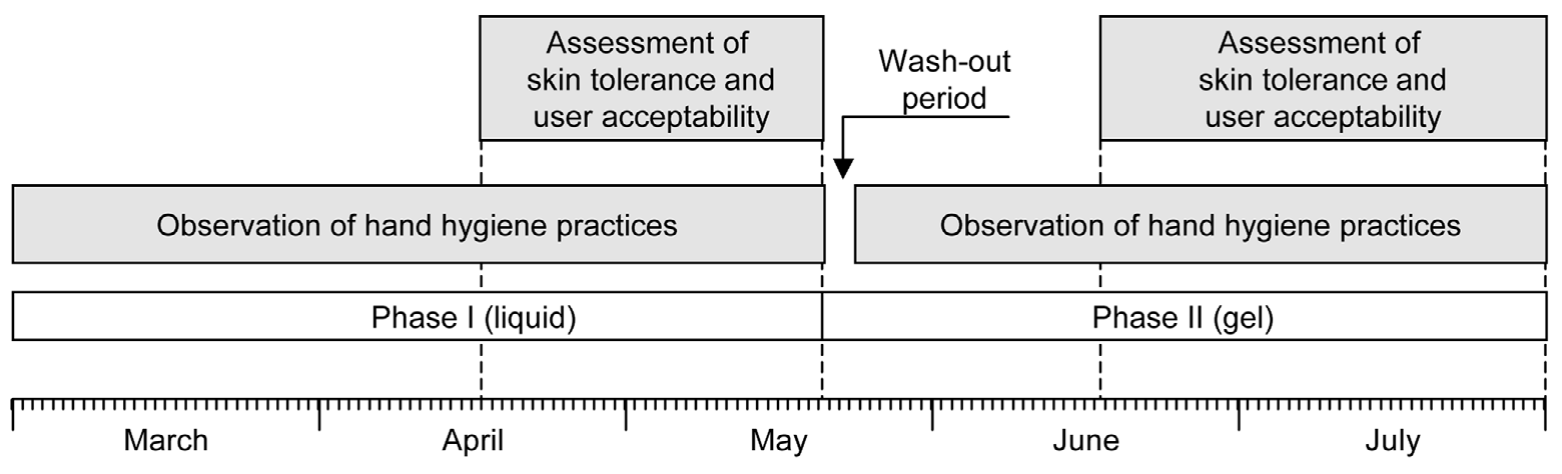
Study design.

During phase I (1 March to 18 May 2004), all ICU staff used the alcohol-based liquid formulation (Hopirub^®^; B. Braun Medical AG, Sempach, Switzerland) in use throughout the institution. During phase II (19 May to 31 July 2004), the liquid was replaced by the gel formulation (Figure 1). Both handrub formulations were widely available in bottles for pocket carriage as well as at different points at the patient bedside [7,14]. The ICU staff comprised 7 physicians, 80 nurses, and 15 nursing assistants throughout the study.

The alcohol-based liquid formulation, Hopirub^®^, has been used extensively in the University of Geneva Hospitals for more than 30 years [7,14]. It contains 75% isopropyl alcohol (wt/wt), 0.5% chlorhexidine gluconate, and isopropyl myristate. The gel formulation used in this study differs from the liquid solution only by the addition of a gelling agent. HCWs were asked not to change their hand-care practices with emollients between phases.

### Compliance with hand hygiene procedures

After formal training and validation during a pilot phase in February 2004, an infection control physician recorded potential opportunities for, and actual performance of, hand hygiene practices during observation sessions distributed equally (Monday to Friday) during both study phases (Figure 1) [7,14]. Patient care activities and indications for hand hygiene were recorded according to standard definitions [1,7,14] on a specially designed report form. Indications comprised activities with a high risk of cross-transmission (for example, before direct patient contact, before invasive contact, and between care of a dirty and a clean body site), medium-risk activities (after patient care), and low-risk activities (indirect patient contact and hospital maintenance). We focused the study on activities with a high risk of cross-transmission.

Hand cleansing was required regardless of whether gloves were put on or changed [1,7,14], and compliance was defined as either washing hands with plain soap and water or rubbing with an alcohol-based formulation. No judgment was made on the quality of the hand-cleansing technique. Potential confounders of hand hygiene compliance included professional category, time of day, patient isolation, accessibility to handrub, and workload as quantified by the number of opportunities for hand cleansing per hour during the observation sessions [7,14,15].

Department chairpersons and ICU staff were informed prior to study initiation. The observer was as unobtrusive as possible but not concealed. The time and location of observation sessions in the ward and the HCWs observed were chosen at random. The observer followed a single HCW in a single- or two-bed space for each individual session as previously described elsewhere [7,14,15]. HCWs could be observed several times throughout the study. No observation was performed during weekends or night shifts. Performance feedback was not given.

### Assessment of skin tolerance

The skin condition of the nurses' and nursing assistants' hands was assessed at the end of each phase (Figure 1) by means of subjective and objective scoring systems and biometric measurements conducted by an independent observer. Larson's Skin Self-Assessment Rating Scale is an ordinal scale with a maximum of 28 points to assess four factors: appearance, integrity, moisture, and skin sensation (7 points for each factor assessed); the lower the score, the worse the skin condition [16]. The Frosch and Kligman [17] observer score rates erythema from 0 to a maximum severity of 4, and wrinkles and desquamation from 0 to 3. Biometric measurements were performed under a Plexiglas hood under controlled environmental conditions (temperature 23°C to 26°C, relative humidity 25% to 45%) after at least 10 minutes of acclimatization. Skin dryness was assessed by the mean value of electrical capacitance measured at three standardized sites on the dominant hand (Corneometer^® ^CM 825; Courage + Khazaka electronic GmbH, Cologne, Germany). Transepidermal water loss (TEWL) (Tewameter^® ^TM 300; Courage + Khazaka electronic GmbH) was measured at three standardized sites on the back of the dominant hand. Participants were asked for a history of atopic and irritative dermatitis. Follow-up by a dermatologist was available at all times.

### Product acceptability

At the end of each study phase, a questionnaire was completed individually by the nurses and nursing assistants (Figure 1). The study focused on the type of care that generated indications for hand hygiene during patient care, and HCWs were asked to give their opinion of and preference for either the liquid or the gel formulation. The following parameters were recorded on a 7-point scale (1 = unpleasant; 7 = pleasant) to obtain an overall acceptability score: color, smell, sticky feeling, irritation, skin dryness, ease of use, speed of drying of the skin after application, and pleasant feeling on application. HCWs were also asked to rate on a 7-point scale to what extent their feeling of being observed during the study phases had modified their compliance with hand hygiene.

### Statistical analysis

Assuming a baseline compliance of 50% with a 0.05 alpha error and 80% power, 530 opportunities in both periods were required to detect a 10% difference in compliance between the liquid and the gel formulations.

First, we performed simple descriptive statistics and compared groups by use of χ^2 ^and non-parametric tests. The unit of analysis was the opportunity for hand hygiene which could be followed or not by handrubbing or handwashing. Logistic regression was used to estimate the association between predictors and compliance and reported odds ratios and 95% confidence intervals. To account for interdependence of observations, we used generalized estimating equations to compute robust estimates of variance and included each HCW as a cluster [7,14]. The main explanatory variable was the hand hygiene formulation, gel versus liquid. Other potential risk factors were evaluated in univariate and multivariate analyses; only variables associated with compliance (*p *< 0.05) were kept in the final multivariate model. The Mann-Whitney *U *test was used to compare the measurement of skin condition and product acceptability score. Two-tailed *p *values of less than 0.05 were considered statistically significant. We used Stata 7.0 (StataCorp LP, College Station, TX, USA) for all analyses.

## Results

From March to July 2004, 379 observation sessions were performed (mean duration, 14.3 ± 8.9 minutes). Characteristics of the observation sessions and opportunities for hand hygiene across the two periods are shown in Table 1. The imbalance in the number of opportunities observed in the morning and afternoon across the study periods was not planned and occurred by chance. There were very few changes in ICU staff during the two periods; during phase II, two nurses and one nursing assistant left the ICU and one nurse was recruited. The proportion of nurses and nursing assistants who believed that they had been observed for hand hygiene compliance was 70% (56/80) and 69% (57/82) during phases I and II, respectively (*p *= 0.95). As calculated by the 7-point self-assessment scale to measure a modification of HCWs' hand-cleansing practice patterns, the mean scores were 3.1 ± 2 and 3.1 ± 1.8 during phases I and II, respectively (*p *= 0.75). Overall, the mean numbers of opportunities per hour were 15.7 ± 9.2 for nurses, 9.6 ± 4.3 for physicians, 10.9 ± 5.8 for nursing assistants, and 13.9 ± 8 for other HCWs.

**Table 1:** Characteristics of the observation sessions and opportunities for hand hygiene across study phases

	Phase I	Phase II	*P *value
Number of observation studies	181	198	
Number of opportunities	604	553	
Median duration in minutes	12.3	13.0	0.74
25%–75% percentiles	8.0–17.0	8.5–16.7	
HCW^a^			0.17
Nurses	132 (73)	134 (68)	
Physicians	29 (16)	29 (14)	
Nursing assistants	12 (6.6)	27 (14)	
Other	8 (4.4)	8 (4)	
Time of day^a^			0.027
Morning	92 (51)	123 (62)	
Afternoon	89 (49)	75 (38)	
Isolated patient^a^	64 (35)	57 (29)	0.14
Availability of handrub^a^			
In the room	177 (98)	197 (99.5)	0.20
In the HCW's pocket	16 (9)	17 (9)	0.53
Median number of opportunities per hour	13.7	11.6	0.004
25%–75% percentiles	8.3–20.6	8.6–16	
Type of opportunity			< 0.001
Respiratory care	40 (6.6)	47 (8.5)	
Intravenous or arterial care	62 (10.3)	60 (10.8)	
Direct patient contact	477 (79.0)	374 (68.0)	
Digestive care	6 (1.0)	12 (2.0)	
Wound care	9 (1.5)	35 (6.0)	
Clean device/material	9 (1.5)	18 (3.2)	
Urinary care	1 (0.2)	7 (1.5)	

^a^Data are presented as numbers (percentages). Phase I: use of liquid handrub; phase II: use of gel handrub. HCW, health care worker.

Compliance varied with type of care: before respiratory tract care, 35.6%; before intravenous or arterial catheter care, 30.3%; before direct patient contact, 34.9%; before digestive tract care, 16.7%; before wound care, 70.4%; before handling clean material, 78%; and before urinary tract care, 25% (*p *< 0.001). A significant difference in compliance was observed across HCW categories: 39.1% among nurses, 27.1% among physicians, 31.1% among nursing assistants, and 13.9% among other HCWs (*p *= 0.027). However, it did not vary according to the intensity of patient care as assessed by the mean number of opportunities per hour (fewer than 10, 10 to 14, more than or equal to 15): 36.4%, 38.4%, and 35.1%, respectively (*p *= 0.14). Compliance was 33.3% (118/354) when the patient was isolated versus 37.8% (304/803) when he/she was not (*p *= 0.23). Compliance improved significantly when the alcohol-based formulation was available in the HCW's pocket: 35.3% (380/1,074) versus 50.6% (42/83) (*p *= 0.035). On average, compliance was higher in the morning than in the afternoon: 41.2% (266/646) versus 30.5% (156/511) (*p *= 0.001).

Table 2 compares compliance with hand hygiene between the two study phases. Overall, compliance increased from 32.1% during phase I to 41.2% during phase II (*p *= 0.035) (Table 2). In multivariate analysis, use of a gel formulation was associated with improved compliance, although the association did not reach statistical significance (Table 3). Importantly, pocket carriage of the alcohol-based handrub was associated with increased compliance. Of note, workload (as estimated by the number of opportunities for hand hygiene per hour) was not a predictor of compliance in multivariate analysis and did not confound the association between study phase and compliance and was therefore removed from the final model.

**Table 2:** Compliance with hand hygiene related to the use of the liquid (phase I) or the gel (phase II) handrub formulation

	Phase I^a^	Phase II^a^	*P *value
Overall compliance	32.1 (194/604)	41.2 (228/553)	0.035
Health care worker category			
Nurses	33.6 (167/497)	45.7 (190/416)	0.011
Physicians	25.8 (16/62)	28.6 (16/56)	0.76
Nursing assistants	32.0 (8/25)	30.8 (20/65)	0.74
Other	15.0 (3/20)	12.5 (2/16)	0.84
Time of day			
Morning	35.0 (106/303)	46.7 (160/343)	0.039
Afternoon	29.2 (88/301)	32.4 (68/210)	0.75
Workload (number of opportunities per hour)			
<10	40.5 (36/89)	33.3 (38/114)	0.29
10–15	39.0 (62/159)	38.0 (89/234)	0.86
≥16	27.0 (96/356)	49.3 (101/205)	< 0.001
Patient isolated			
Yes	32.0 (65/203)	35.1 (53/151)	0.9
No	32.2 (129/401)	43.5 (175/402)	0.017
Availability of handrub			
Yes	46.9 (23/49)	55.9 (19/34)	0.53
No	30.8 (171/555)	40.3 (209/519)	0.035
Availability of alcohol-based solution in the room			
Yes	32.3 (193/598)	41.0 (224/547)	0.05
No	16.7 (1/6)	66.7 (4/6)	0.068
Type of opportunity			
Respiratory care	40 (16/40)	31.9 (15/47)	0.56
Intravenous or arterial care	24.2 (15/62)	36.7 (22/60)	0.17
Direct patient contact	31.7 (151/477)	39.0 (146/374)	0.13
Digestive tract care	0 (0/6)	25 (3/12)	NA
Wound care	55.6 (5/9)	74.3 (26/35)	0.24
Clean device	77.8 (7/9)	77.8 (14/18)	0.87
Urinary care	0 (0/1)	28.6 (2/7)	NA

^a^Data not in parentheses are presented as percentages. NA, not applicable.

**Table 3:** Independent factors associated with compliance with hand hygiene

Variable	Odds ratio (95% CI)	*P *value
Phase		
Liquid formulation	1	
Gel formulation	1.33 (0.97–1.82)	0.072
Time of day		
Morning	1	
Afternoon	0.61 (0.45–0.84)	0.002
Health care worker category		
Nurses	1	
Physicians	0.61 (0.37–1.01)	0.053
Nursing assistants	0.51 (0.28–0.92)	0.024
Other	0.30 (0.11–0.82)	0.020
Pocket carriage of handrub		
No	1	
Yes	1.86 (1.06–3.28)	0.031
Type of opportunity		
Respiratory care	1	
Intravenous or arterial care	0.85 (0.47–1.53)	0.60
Direct patient contact	1.16 (0.73–1.86)	0.534
Digestive care	0.41 (0.12–1.39)	0.151
Wound care	3.13 (1.40–7.02)	0.005
Clean device/material	7.25 (2.60–20.19)	< 0.001
Urinary care	0.54 (0.10–3.06)	0.488

CI, confidence interval.

Eighty HCWs (66 nurses and 14 nursing assistants) participated in the skin tolerance evaluation during phase I, and 82 (68 nurses and 14 nursing assistants) during phase II (Table 4). Mean user acceptability scores for the liquid and gel formulations were 39.1 ± 7.3 and 40 ± 7.6, respectively (*p *= 0.44). Based on self-assessment, observer assessment, and the measurement of epidermal water content, the gel performed significantly better than the liquid formulation (Table 4). At the end of phase II, 47 HCWs (57%) rated the gel as better than the liquid formulation, 13 (16%) as equivalent to the liquid formulation, whereas 22 (27%) considered the gel formulation to be inferior.

**Table 4:** Evaluation of skin condition by clinical scores and instrument measurements

	Phase I	Phase II	*P *value
Age (years)^a^	36.8 ± 8.8	36.0 ± 9.1	0.57
Female	80% (64/80)	77% (63/82)	0.62
Self-assessment score^a^	17.1 ± 6.2	21.2 ± 7.2	0.001
Observer assessment score^a^	1.5 ± 1.8	0.5 ± 0.8	< 0.001
Epidermal water content^a^	20.7 ± 6.2	25.1 ± 7.1	< 0.001
Epidermal water loss (g/m^2 ^per hour)^a^	19.8 ± 9.3	20.4 ± 8.2	0.66

^a^Values are presented as mean ± standard deviation.

## Discussion

Evaluation of the effect of a gel versus a liquid formulation on hand hygiene adherence and skin health is a key issue [8,9]. Personal comfort and the likelihood that better tolerance will lead to better product acceptance and improved compliance support the importance of a healthy skin barrier [8,9]. Because the liquid and gel formulations tested differed only by the addition of a gelling agent, observed differences in our study cannot be associated with the active ingredient.

Prospective observation of hand hygiene by a single observer is the most accurate means to assess compliance, and a comprehensive evaluation of skin condition was also performed. Overall compliance with hand hygiene recommendations was rather low but within the range observed in other studies [9], particularly those that were conducted in critical care and that focused on indications before patient care or contact [9,14,15,18]. As previously reported, compliance varied according to HCW category and was lower among physicians than nurses [7,14,18]. Introduction of the gel formulation was associated with improved compliance among nurses but not among physicians, nursing assistants, and other HCWs. Importantly, immediate access to alcohol-based handrubs was the stronger predictor of compliance. Availability of a handrub at the point of care, whether liquid or gel, increased compliance independently of the type of formulation, time of day, professional category, and other confounders.

Reported reasons for poor HCW compliance with hand hygiene include skin irritation [8,19,20]. This issue is of particular relevance in critical care, where the need for hand hygiene is high [14,18]. Our results show that the acceptability of the gel formulation was very high and that HCWs' skin condition improved during the study phase when the gel formulation was in use. No case of significant skin damage was observed for either product. Sustainability is a critical issue of hand hygiene promotion strategies, and user acceptability and skin tolerance of handrubs are key enabling factors [7,9,21].

Self- and observer-assessment variables as sensitive and reliable indicators of product acceptability were similar to those in earlier studies [16,21]. All studies on skin tolerance, whether performed with volunteers or HCWs, showed that alcohol-based formulations, either liquid or gel, were much better tolerated than handwashing with antimicrobial or non-antimicrobial soap. Our study suggests that the gel formulation tested is better tolerated than the corresponding liquid formulation. Studies of the effects of alcohol-based products on HCWs' skin have been performed on volunteers with normal skin in a non-clinical setting [21-24]. They were, therefore, less representative of field conditions that are characterized by the multifactorial interplay between skin integrity, skin flora, and individual attitudes [25-28].

Our study was not designed to evaluate the microbiological effectiveness of the gel formulation. Although the limited efficacy of some alcohol-based hand gels has been reported [22,29], the gel formulation used in this study, in contrast to earlier test products [29], meets the European Norm (EN)1500 standard for alcohol-based handrubs within 30 seconds.

There are several limitations to this study. First, because the HCWs and the observer were not blinded, assessments could have been biased. However, the Corneometer^® ^and the TEWL allowed objective measurement. Furthermore, except for TEWL data that did not show a difference between the two phases, all methods used to assess HCWs' skin hand condition yielded concordant results. Similarly, Winnefeld and colleagues [25] reported that TEWL was less sensitive than self-assessment at detecting a difference in skin tolerance. Second, HCWs may have changed their behavior because they were being observed. Observation bias is likely to increase compliance estimates. However, in each study phase, a similar proportion of HCWs felt that they had been observed, thus arguing against a major impact of observation on overall results [30]. Also, we cannot rule out that the improvement in compliance was due to the availability of a new formulation with a Hawthorne effect. Only repeated observations at a later time point could demonstrate a sustained improvement, but unfortunately, the observations in this study could not be extended longer than twonths following the change of formulation. Importantly, both the use of the gel formulation and the facilitated access to a handrub independently predicted improved compliance. Furthermore, considering the excellent tolerance of both formulations and user preferences, both formulations are currently proposed to ICU staff. Third, seasonal variation may have contributed to the better skin condition observed during phase II (between May and July), when the gel formulation was used, although only very low temperatures have been shown to have a significant impact on skin condition [31]. Fourth, generalizability of study results requires additional testing among other HCW populations, in other health care settings, and with other handrubs. Finally, the study was not powered to assess differences in infection rates.

## Conclusion

This is the first study to compare the use of an alcohol-based liquid versus a gel formulation on hand hygiene compliance in daily patient care. The gel was associated with better skin condition, superior acceptance, and a trend toward improved compliance as compared to the liquid formulation. Immediate access to the handrub was the strongest predictor of compliance. Whether our study findings are generalizable to other gel formulations and whether the observed improvement in hand hygiene would result in a decrease in health care-associated infection remain to be determined.

## Key messages

• HCWs' hands play a key role in the patient-to-patient transmission of microbial pathogens.

• Alcohol-based handrubs are the new standard for hand hygiene action worldwide.

• Good dermal tolerance and user acceptability of handrub products are essential pre-requisites for an improvement of hand hygiene practices in health care.

• This is the first study comparing the use of an alcohol-based liquid handrub versus a gel formulation on hand hygiene compliance in daily patient care.

• Immediate access to the handrub is the strongest predictor of better compliance.

## Abbreviations

HCW = health care worker; ICU = intensive care unit; TEWL = transepidermal water loss.

## Competing interests

The authors declare that they have no competing interests.

## Authors' contributions

DP and SH developed the study design and coordinated its implementation. OT coordinated the study implementation, was responsible for data collection, and drafted the manuscript. JL coordinated the dermal scoring systems and biometric measurements. WG developed the gel formula and provided intellectual content. All authors read and approved the final manuscript.
